# Diversity of Killer Cell Immunoglobulin-Like Receptor (KIR) Genotypes and KIR2DL2/3 Variants in HCV Treatment Outcome

**DOI:** 10.1371/journal.pone.0099426

**Published:** 2014-06-13

**Authors:** Jose Ramón Vidal-Castiñeira, Antonio López-Vázquez, Jesús Martínez-Borra, Pablo Martínez-Camblor, Jesús Prieto, Rosario López-Rodríguez, Paloma Sanz-Cameno, Juan de la Vega, Luis Rodrigo, Rosa Pérez-López, Ramón Pérez-Álvarez, Carlos López-Larrea

**Affiliations:** 1 Immunology Service, Hospital Universitario Central de Asturias, Oviedo, Spain; 2 Oficina de Investigación Biosanitaria (OIB), Oviedo, Spain; 3 Liver Unit and Division of Hepatology and Gene Therapy, Clínica Universitaria de Navarra, University of Navarra, Pamplona, Spain; 4 Liver Unit, Gastroenterology Service, Hospital Universitario de La Princesa, Instituto de Investigación Sanitaria Princesa (IIS-IP), Universidad Autónoma de Madrid and CIBERehd, Instituto de Salud Carlos III, Madrid, Spain; 5 Gastroenterology Service, Hospital San Agustín, Avilés, Spain; 6 Gastroenterology Service, Hospital Universitario Central de Asturias, Oviedo, Spain; 7 Fundación Renal Iñigo Álvarez de Toledo, Madrid, Spain; Karolinska Institutet, Sweden

## Abstract

The aim of this study was to analyse the distribution of KIR haplotypes and the *KIR2DL2/3* alleles in chronic HCV-infected patients in order to establish the influence on the response to pegylated interferon plus ribavirin classical treatment. The alleles study of previously associated *KIR2DL2/3* showed that KIR2DL2*001 was more frequent in non-SVR (NSVR) (42.2% vs. 27.5%, p<0.05) and KIR2DL3*001 was associated with sustained viral response (SVR) (41.6% vs. 61.2%, p<0.005). The KIR2DL3*001-HLA-C1 association was also significant (24.5% vs. 45.7%, p<0.001). From the frequencies of KIR obtained, 35 genotypes were assigned on the basis of previous studies. The centromeric A/A genotype was more frequent in SVR (44.1% vs. 34.5%, p<0.005) and the centromeric B/B genotype was found to be significantly more frequent in NSVR (20.9% vs. 11.2%, p<0.001). The logic regression model showed the importance of KIR genes in predicting the response to combined treatment, since the positive predictive value (PPV) was improved (from 55.9% to 75.3%) when the analysis of KIR was included in addition to the *IFNL3* rs12979860 polymorphism. The study of KIR receptors may be a powerful tool for predicting the combined treatment response in patients with chronic HCV infection in association with the determination of *IFNL3* polymorphism.

## Introduction

Innate immunity is the first line of defence against pathogens, and operates non-specifically [1]. Natural killer (NK) cells are important effector lymphocytes that participate in this early immune response to pathogens, including virally infected cells, and tumoral cells through the production of cytokines and chemokines [2]. NK cell function is regulated by a network of activating and inhibitory receptors [3], including killer immunoglobulin-like receptors (KIRs) [4].

KIRs, which are members of the CD158 gene family, are clustered in a 160 kilobase (kb) length of the 19q13.4 chromosome region within the leukocyte receptor complex (LRC) [5]. This diverse family of activating and inhibitory receptors modulates the development and activity of NK cells and some subpopulations of CD8+ T cells by interacting with the class I major histocompatibility complex (MHC) [6]. Although this recognition is well established, their interactions have yet to be fully understood. Genetic analyses indicate that KIR variation, in conjunction with polymorphic MHC class I genes, plays a key role in immune defence [7]. Thus, the extensive polymorphisms of HLA and KIR genes and their independent segregation give rise to unusual expression features. KIR receptors for which there is no HLA ligand can be expressed, while conversely an HLA ligand can be expressed for which there is no KIR. Furthermore, the interactions are influenced by peptides that bind to HLA class I and are contacted by KIR [8].

To date, 14 KIR receptors and two pseudogenes have been identified, and on the basis of this variation in gene content, more than 50 KIR haplotypes have been identified [9].

Previous analyses of these haplotypes indicate that they are subdivided into two groups: haplotype A and haplotype B [10]. Group A haplotypes comprise seven genes (*KIR2DL1, KIR2DL3, KIR2DL4, KIR3DL1, KIR3DL2, KIR3DL3* and *KIR2DS4*) and two pseudogenes (*KIR2DP1* and *KIR3DP1*). The gene *KIR2DS4* is potentially activating but is disabled by a 22-bp frameshift deletion in approximately 75% of A haplotypes and is only functional in a minority of individuals [11]. Moreover, *KIR2DL4* encodes a receptor that has both inhibitory and activating functions [12]. In contrast, Group B haplotypes are composed of varying numbers of KIR genes, including at least one of the following KIRs: *KIR2DL2*, *KIR2DL5, KIR3DS1, KIR2DS1, KIR2DS2, KIR2DS3* and *KIR2DS5*. Subsequently, these two groups were shown to have distinct associations with several diseases [13], [14]. Classic linkage disequilibrium (LD) studies identified two distinct regions in the KIR cluster around the *KIR2DL4* gene: a centromeric (cen) region, which seems to be driven by the *KIR2DL5* and *KIR2DL2/*3 loci [15], and a telomeric (tel) region driven by the *KIR3DL1/S1* locus [10].

Several studies have shown that individuals vary in the number and type of KIR loci they contain [16]. This variability and biomedical relevance of KIRs make it important to study their organization. There is increasing evidence that receptor–ligand specificity between polymorphic KIRs and polymorphic MHC class I genes is associated with a wide range of infections, such as HIV and hepatitis C, in addition to autoimmune diseases [17], disorders in pregnancy [18], and in bone marrow and solid organ transplantation [19], [20], [21].

Hepatitis C virus (HCV) is a hepatotropic non-cytopathic positive-strand RNA virus that is a major cause of chronic hepatitis, cirrhosis, and hepatocellular carcinoma, affecting over 170 million people worldwide [22]. Pegylated interferon-alpha (Peg-IFN-α) plus Ribavirin has been the most effective therapy for chronic hepatitis C [23], until the emergence of new directly acting antiviral agents (DAA) like Boceprevir and Telaprevir. Several host and viral factors influence the treatment outcome [24], [25] and approximately 50% of patients with HCV genotype 1 achieve a sustained viral response (SVR) [26]. Recently, single nucleotide polymorphisms (SNPs) in the *IFNL3* gene (Also known as *IL28B*) that influence the responsiveness of treatment of HCV infection and the clearance of HCV have been identified [27], [28]. The strongest association has been shown with the *IFNL3* SNP rs12979860 C/C genotype, which has an SVR ratio over 70% in European-American and Hispanic patients with the HCV genotype 1 [27]. Other host genetic factors associated with the response outcome have been reported, such as KIR [29] and the PD-1.3 polymorphism in the *PDCD1* gene, which has recently been described by our group [30].

The aim of this study was to investigate the role of KIR genes and their genotypes on the response to combined therapy in a well-characterised group of patients with chronic HCV infection. We also examined the effect of the different *KIR2DL2/2DL3* alleles and KIR haplotypes on the response outcome. Finally, a mathematical model was developed to evaluate the combined effect of *IFNL3* polymorphisms and KIR receptors.

## Materials and Methods

### Study population

A cohort of 811 unrelated HCV genotype 1 infected Caucasian patients was recruited from several Spanish Hospitals: Hospital Universitario Central de Asturias (HUCA), Hospital San Agustín (Aviles), Clínica Universitaria de Navarra (CUN) and Hospital La Princesa (Madrid).

Patients included in this study had received their first course of antiviral therapy. The duration of treatment with PEG-IFN-α-2a or α-2b and Ribavirin was established in 48 weeks on the basis of the HCV genotype, according to consensus clinical guidance. Those without HCV RNA in their sera by six months post-treatment were defined as sustained viral responders (SVR, n = 313). The other patients were defined as non-sustained viral responders (NSVR, n = 498) which included non-responders, relapsers and partial responders.

This study was granted ethical approval by the Regional Ethics Committee for Clinical Investigation of all hospitals (Regional Ethics Committee of Clinical Research of *Principado de Asturias*; Ethics Committee of Clinical Research of *Navarra*; Regional Ethics Committee of Clinical Research of *Madrid*). All participants provided written informed consent.

### DNA extraction

Genomic DNA was extracted from peripheral blood with the Magtration-MagaZorb DNA Common Kit-200 N using the Magtration 12GC system (Precision System Science Co., Ltd., Woerrstadt, Germany) and the Maxwell 16 Blood Purification Kit using the Maxwell 16 Instrument (Promega Corporation, Madison, Wisconsin, USA).

### IFNL3 genotyping

The rs12979860 SNP was genotyped by amplifying the region containing the polymorphic site and hybridisation with fluorescent-labelled probes in an RT-PCR based on the melting-curve analysis using the Light-Cycler system (Roche Diagnostics, Mannheim, Germany) [30].

### HLA/KIR genotyping

The HLA-B, HLA-C, and KIR genes were typed by using Lifecodes HLA-SSO and KIR-SSO typing kits (Tepnel Lifecodes Corporation, Stamford, UK) based on the Luminex Multi-Analyte Profiling system (xMAP technology) (Luminex Corp., Austin, TX), following the manufacturer's instructions. Ambiguities in KIR typing were resolved by PCR-single specific primer (SSP), as defined previously [31]. HLA-Bw4, HLA-Bw6, HLA-C1 and HLA-C2 were assigned on the basis of the amino acid residues of HLA-B and HLA-C alleles [32]. The KIR genotypes were deduced from KIR profiles as previously described [10], [33]. KIR gene profiles were classified with respect to the centromeric and telomeric regions of the KIR A and B haplotypes: Cen-A driven by *KIR2DL3* while Cen-B by *KIR2DL2*. On the other hand the Tel-A is driven by *KIR3DL1* and Tel-B by *KIR3DS1*.

Finally, a PCR was also designed for *KIR2DL5* subtyping to distinguish between expressed and non-expressed variants of this gene, as previously described [34].

### *KIR2DL2* and *KIR2DL3* oligotyping

Based on the results of KIR genotyping, patients with *KIR2DL2* and/or with *KIR2DL3* genes were genotyped as described elsewhere [35], [36] to identify different alleles. 146 NSVR and 103 SVR patients were randomly selected for this study. Different regions of these KIR receptors were selectively amplified by a PCR using locus-specific primers. For SSOP hybridisation, amplified DNA was blotted onto nylon membranes and hybridised with a panel of 5 and 13 SSOPs designed to detect unique sequence motifs of known *KIR2DL2* and *KIR2DL3* alleles, respectively. Alleles were assigned with respect to the reaction patterns of the SSOP, based on the known *KIR2DL2* and *KIR2DL3* sequences. This method identifies the most common alleles for *KIR2DL2* (*001, *002, *003, *004 and *005) and for *KIR2DL3* (*001, *002, *003, *004, *005 and *006).

### Statistical analysis

Continuous variables were summarised as the mean and standard deviation. Categorical variables were summarised as absolute frequencies and percentages. The Genetic Analysis Package (GAP) in R (www.r-project.org) was used to compute LD statistics for pairs of alleles. In particular, the D' statistic based on Cramer's V was used. The general bootstrap algorithm (gBA) [37] was used to compare global and particular LDs in responders and non-responders. Fisher’s exact test was used to examine the relationship between categorical variables. In addition, where the relationships were statistically significant (p < 0.05) the odds ratios (ORs) and the respective 95% confidence intervals were also reported. Finally, the R package LogicReg (available in the CRAN) was used to develop a logic regression (LR) model [38]. A stepwise criterion based on the Akaike interaction was included in this model. LR aims to identify predictors that are Boolean (logical) combinations of the original predictors. In particular, LR looks for an optimal model of the form: 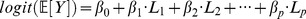
where Y is the response (Yes/No), *β_1_,…, β_p_* are the parameters, and *L_1_,…, L_p_* are Boolean combinations (intersection (AND, 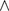), unions (OR 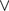) and complement (NOT, ^C^) of the dichotomous variables considered. The *L_i_*'s are also called logic trees. Our model considered the variables: *IFNL3* rs12979860 genotype, *KIR2DL2/3*, *KIR2DL2-HLA*, *KIR2DL3-HLA*, KIR Genotype, KIR centromeric motif, KIR telomeric motif and *KIR2DL2/3* alleles described in this study. We also included clinical parameters like viral load, BMI, sex, etc. The logic trees were selected by a greedy search based on the usual deviance as the measure of model fit. Finally, the ROC curve and the area under the ROC curve (AUC) were used to check the quality of the model obtained.

## Results

### Distribution of KIR receptors frequency

A total of 313 patients (38.4%) with SVR and 498 (61.4%) with NSVR were recruited for the study. All of them were genotyped for HLA-B, HLA-C and KIR receptors. As shown in table 1, the framework genes *KIR2DL4, KIR3DL2, KIR3DL3* and the pseudogene *KIR3DP1* were present in all individuals. Also, as previously described [29], [39], three KIR genes were associated with chronic hepatitis C treatment response: *KIR2DL2* was more frequent in the NSVR group (OR = 1.51 95%, CI = 1.13–2.02; p<0.005) while *KIR2DL3* was associated with good treatment outcome (OR = 0.48, 95% CI = 0.32–0.72; p<0.001). On the other hand, the activating *KIR2DS2* gene was also associated with a poor response to combined therapy (OR = 1.55, 95% CI = 1.16–2.07; p<0.005). Moreover, considering *KIR2DL2* and *KIR2DL3* alleles of the same locus [15], the differences between the two groups of patients were statistically significant when they were in the homozygous state: *KIR2DL2/KIR2DL2* with NSVR (20.7% vs. 11.2%, p<0.001) and *KIR2DL3/KIR2DL3* with SVR (35.3% vs 45%, p < 0.01). Differences in other KIR receptors studied were not significant.

**Table 1:** KIR gene frequencies, *KIR2DL2/3-HLA* and *KIR2DS2-HLA* genotypes distribution in the two groups of patients.

	NSVR (%)n = 498	SVR (%)n = 313	OR (95% CI)	p
**KIR2DS1**	210 (42.2)	130 (41.5)		
**KIR2DS2**	317 (63.7)	166 (53)	1.55 (1.16–2.07)	<0.005
**KIR2DS3**	200 (40.2)	116 (37.1)		
**KIR2DS4**	462 (92.8)	295 (94.2)		
**KIR2DS5**	176 (35.3)	111 (35.5)		
**KIR2DP1**	468 (94)	302 (96.5)		
**KIR2DL1**	466 (93.6)	302 (96.5)		
**KIR2DL2**	323 (64.9)	172 (55)	1.51 (1.13–2.02)	<0.005
**KIR2DL3**	394 (79.1)	278 (88.8)	0.48 (0.32–0.72)	<0.001
**KIR2DL4**	498 (100)	313 (100)		
**KIR2DL5A**	212 (42.6)	137 (43.8)		
**KIR2DL5B**	176 (35.3)	97 (31)		
**KIR3DL1**	461 (92.6)	290 (92.7)		
**KIR3DL2**	498 (100)	313 (100)		
**KIR3DL3**	498 (100)	313 (100)		
**KIR3DP1**	498 (100)	313 (100)		
**KIR3DS1**	211 (42.4)	136 (43.5)		
**KIR2DL2/KIR2DL2**	104 (20.7)	35 (11.2)	2 (1.31–3.03)	<0.001
**KIR2DL2/KIR2DL3**	219 (44)	137 (43.8)	-	
**KIR2DL3/KIR2DL3**	175 (35.3)	141 (45)	0.66 (0.49–0.88)	<0.01
**KIR2DS2-HLAC1**	255 (51.2)	128 (40.9)	1.46 (1.09–1.96)	<0.01
**HOMOKIR2DL2-HLAC1C1**	32 (6.4)	13 (4.1)	-	
**HOMOKIR2DL2-HLAC1C2**	61 (12)	14 (4.4)	2.85 (1.56–5.26)	<0.001
**HOMOKIR2DL2-HLAC2C2**	11 (2.2)	8 (2.6)	-	
**KIR2DL2/2DL3-HLAC1C1**	74 (14.9)	49 (15.7)	-	
**KIR2DL2/2DL3-HLAC1C2**	95 (19)	58 (18.5)	-	
**KIR2DL2/2DL3-HLAC2C2**	50 (10.1)	30 (9.9)	-	
**HOMOKIR2DL3-HLAC1C1**	32 (6.4)	51 (16.3)	0.34 (0.21–0.54)	<0.001
**HOMOKIR2DL3-HLAC1C2**	91 (18.5)	60 (19.2)	-	
**HOMOKIR2DL3-HLAC2C2**	52 (10.5)	30 (9.3)	-	

Next, we have genotyped for HLA-B and HLA-C alleles but we did not find statistical differences except for HLA-C*07. We found that this allele, which belonged to HLA-C1 group, was more frequent in patients with SVR (2n = 148, 23.6%) than in non responders (2n = 171, 17.2%) (p<0.005, Table S1).

When the distribution of KIR inhibitory receptors with their HLA ligands was analysed, we found, as previously described, that the homozygous *KIR2DL3-HLA-C1* genotype was significantly more common in patients with SVR than in those with NSVR (OR = 0.34, 95% CI = 0.21–0.54; p<0.001). In contrast, *KIR2DL2-HLA-C1C2* was more frequent in NSVR patients (OR = 2.85, 95% CI = 1.56–5.26; p<0.001). We also found that KIR2DL3-HLA-C*07 was significantly associated with a good treatment outcome (27.3% in NSVR vs 45% in SVR, p<0.001, table S1).

As expected, with regard to *KIR2DS2*, we observed an association of *KIR2DS2-HLA-C1* with NSVR (51.2% vs. 40.9%; p<0.01). No other significant association was found in relation to *KIR2DS2*. Finally, we did not find statistical associations between *KIR3DL1/S1+HLA-Bw480I/80T* genotypes and treatment outcome. In fact, *KIR3DS1*, previously associated with hepatocellular carcinoma (HCC) progression [40], was also not significant.

### Oligotyping of KIR2DL2 and KIR2DL3

Having found an association of *KIR2DL2* and *KIR2DL3* with treatment outcome, we decided to analyse the possible influence of different *KIR2DL2/KIR2DL3* alleles in the response to HCV treatment (Table 2). For *KIR2DL2*, five alleles were analysed (KIR2DL2*001-005), but only KIR2DL2*001 and KIR2DL2*003 were identified in our cohort. In the case of *KIR2DL3*, six alleles were analysed (KIR2DL3*001-006), with only KIR2DL3*001 and KIR2DL3*002 being found in our population.

**Table 2:** Genotyping and frequencies of *KIR2DL2* and *KIR2DL3* alleles in relation to treatment outcome.

	NSVR(n = 146)	SVR(n = 103)	p	OR (95% CI)
KIR2DL2*001	61 (41.8)	28 (27.2)	<0.05	1.92(1.11–3.31)
KIR2DL2*003	38 (26)	31 (30.1)	-	-
KIR2DL3*001	60 (41.1)	63 (61.2)	<0.005	0.44 (0.26–0.74)
KIR2DL3*002	74 (50.7)	45 (43.7)	-	-
KIR2DL3*001-HLA-C1	35 (24)	47 (45.6)	<0.001	0.37(0.22–0.65)
KIR2DL3*002-HLA-C1	60 (41.1)	37 (35.9)	-	-

The study of the contribution of these alleles to the treatment response showed that the KIR2DL2*001 was significantly more frequent in NSVR (OR = 1.92, 95% CI = 1.11–3.31; p<0.05) and the KIR2DL3*001 was associated with SVR (OR = 0.44, 95% CI = 0.26–0.74; p<0.005). Moreover, KIR2DL3*001 in combination with HLA-C1 ligand was related to a good treatment outcome (OR = 0.37, 95% CI = 0.22–0.65; p<0.001). No association was found between KIR2DL3*002-C1, KIR2DL2*001-C1 and KIR2DL2*003-C1 (data not shown).

### KIR haplotype frequency in patients with chronic HCV infection

**Figure 1:**
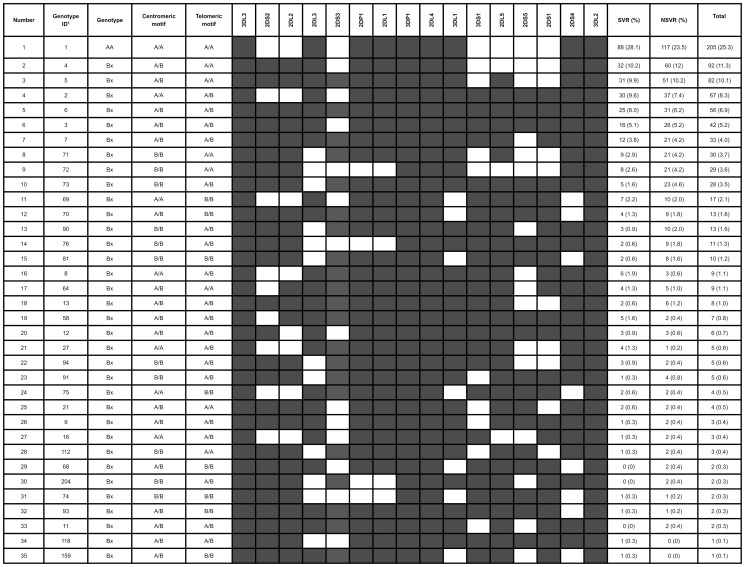
KIR genotype distribution in the study cohort. The genotypes were deduced from KIR profiles as previously described [10], [33]. Note: ^1^ID assigned by the Allele Frequency Net Database (http://www.allelefrequencies.net)

In the current study, the KIR genotypes were analysed to determine the KIR haplotypes, based on those characterised in other studies [10], [33]. Thirty-five genotypes were identified in this cohort and were resolved into corresponding pairs of haplotypes (Figure 1).

The most common genotype was *KIR3DL3-2DL3-2DP1-2DL1-3DP1-2DL4-3DL1-2DS4-3DL2* (Genotype N° 1), corresponding to homozygosity for the major subtype of the previously reported *A* haplotype (28.1% in SVR and 23.5% in NSVR). Most of these genotypes included the KIR2DL3*001 allele. The other KIR genotypes found included at least one activating locus in addition to *KIR2DS4*, corresponding to haplotype B. These genotypes are named Bx. In general, we observed that genotypes that included *KIR2DL2*, especially the *001 allele, were present at a higher frequency in NSVR patients. The most prevalent was *KIR3DL3-KIR2DS2-KIR2DL2-2DL3-2DP1-2DL1-3DP1-2DL4-3DL1-2DS4-*3DL2 (genotype N° 2), which includes the *KIR2DS2* in strong LD with the *KIR2DL2* gene. On the other hand, in SVR patients, the predominant genotypes were those that included *KIR2DL3*, like the aforementioned genotype 1.

**Figure 2:**
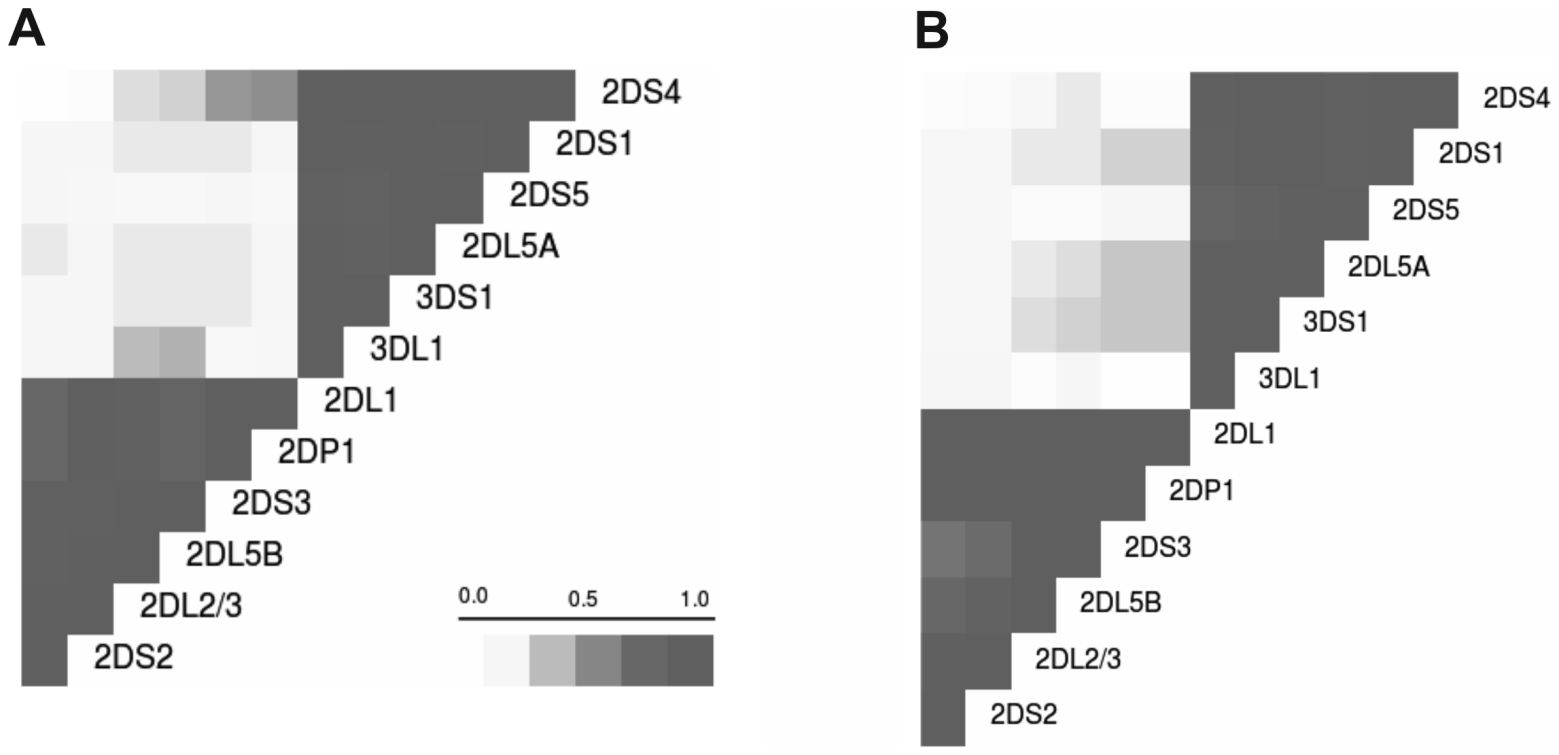
Pairwise D' LD based on Cramer's V correlation coefficient between the presence and absence of different KIR genes in the two groups of patients (A: Non Sustained Viral Responders, NSVR; B: Sustained Viral Responders, SVR). In this approach to assessing KIR LD, the KIR cluster genetic polymorphism is considered simply as the presence (POS) or absence (NEG) of KIR genes. Note: Several conditions were applied for this analysis: 1) *KIR3DL3, KIR3DP1, KIR2DL4* and *KIR3DL2* were not included because they were present in all patients (framework genes), 2) *KIR2DL2* and *KIR2DL3* were considered as alleles of the same locus, 3) *KIR2DL5* was differentiated in *KIR2DL5A* and *KIR2DL5B* because they are located at different positions, and 4) *KIR2DS3* was included in the centromeric region.

The overall patterns of linkage disequilibrium of KIR receptors were similar in two groups of patients (Figure 2). As previously described, the measures showed two distinct regions in the KIR cluster around the KIR2DL4 gene. One was a centromeric region (*KIR3DL3, KIR2DS2, KIR2DL2, KIR2DL3, KIR2DS3, KIR2DP1, KIR2DL1* and *KIR3DP1*) that appeared to be driven by the *KIR2DL5* and *KIR2DL2/3* locus; the other was a telomeric region (*KIR2DL4, KIR3DL1, KIR3DS1, KIR2DL5, KIR2DS5, KIR2DS1, KIR2DS4* and *KIR3DL2*), driven by *KIR3DL1/S1*.

Subdividing the KIR cluster into the two regions identified by the LD study, simplifies the description of the haplotype structure of the KIR genes. When we compared the frequency of centromeric and telomeric motifs in the two groups, we found that the Cen-A haplotype in homozygous was significantly more common in SVR patients (OR = 0.63, 95% CI = 0.47–0.84; p<0.005) and the Cen-B haplotype segment in homozygous was more frequent in NSVR patients (OR = 2.04, 95% CI = 1.35–3.03; p<0.001) (Table 3). Other combinations were not significant. Finally, we analysed the association of the centromeric and telomeric KIR haplotypes with HLA-C and HLA-B. We found a statistically significant association of the Cen-A/A motif and HLA-C with good treatment outcome (OR = 0.62, 95% CI = 0.45–0.84; p<0.005). In contrast, the association of the Cen-B/B motif and HLA-C combination was significant (OR = 2.5, 95% CI = 1.5 –3.96; p<0.001) in relation with non-response. The Tel-HLA combinations were not found to be associated with treatment outcome.

**Table 3:** KIR genotypes and haplotype frequencies of the studied population, and combinations of Cen and Tel haplotypes with HLA-B and HLA-C.

	NSVR (n = 498)	SVR (n = 313)	OR (95% CI)	p
**Genotype**				
**AA**	117 (23.5)	88 (28.1)		NS
**Bx^1^**	381 (76.5)	225 (71.9)		
**Centromeric** **haplotype segment**				
**A/A**	172 (34.5)	138 (44.1)	0.63 (0.47–0.84)	<0.005
**A/B**	222 (44.6)	140 (44.7)	-	NS
**B/B**	104 (20.9)	35 (11.2)	2.04 (1.35–3.03)	<0.001
**Telomeric haplotype segment**				
**A/A**	279 (56)	133 (55.3)		NS
**A/B**	176 (35.3)	115 (36.7)		
**B/B**	43 (8.6)	25 (8)		
**Cen-HLA combinations**				
**Cen-A/A-HLA-C1**	121 (24.3)	107 (34.2)	0.62 (0.45–0.84)	<0.005
**Cen-A/B-HLA-C1**	172 (34.5)	110 (35.1)	-	NS
**Cen-B/B-HLA-C1**	92 (18.5)	26 (8.3)	2.5 (1.58–3.96)	<0.001

**Note**: ^1^B/B and A/B genotypes were included in this group.

Tel-HLA-C, Cen-HLA-B and Cen-HLAB combinations were not significant.

### Analysis of KIR genotyping and *IFNL3* polymorphism combinations

**Figure 3:**
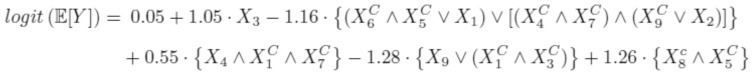
Note: Cen-AA (X1), Cen-BB (X2), IFNL3-G/G (X3), IFNL3-G/T (X4), IFNL3-T/T (X5), HOMOKIR2DL3-C1C1 (X6), HOMOKIR2DL2-C1C1 (X7), HOMOKIR2DL2-C1C2 (X8) viral load less than 400000 UI/ml (X9).

We used logic regression to examine Boolean combinations (AND/OR/NOT) of binary covariates considered in this study, in order to derive a model for predicting chronic HCV treatment outcome. The final model obtained is summarised as follows. Combinations of KIR genotyping, *IFNL3* and the intersections and unions of other factors can be expressed mathematically (figure 3).

The measure of the importance of a variable in the response to treatment is the frequency with which it appears independently in all models. From the summary, the contribution of each variable can be evaluated by the ratio of the number of times it was included in any of the models divided by the total number of models.

**Figure 4:**
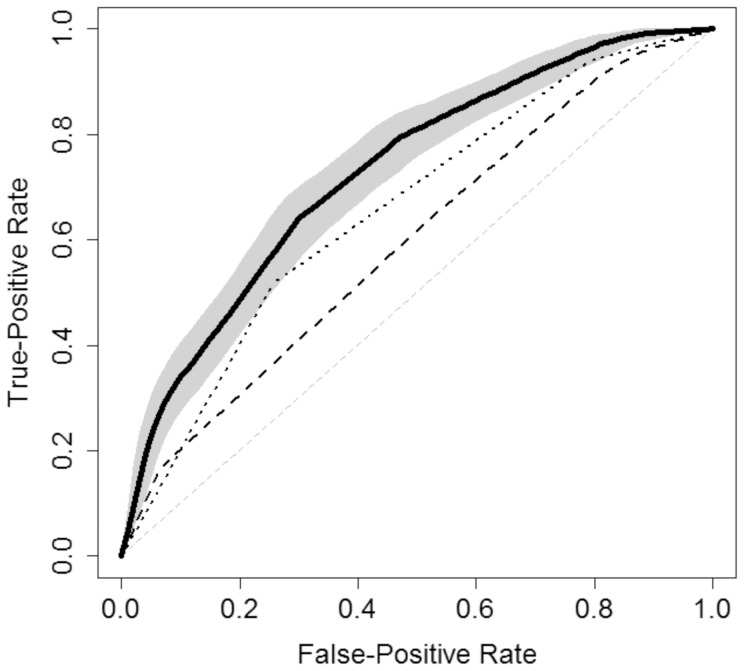
ROC curve of logic regression model using *IFNL3* C/C and KIR/HLA gene determination (*KIR2DL2/3*, *KIR2DL2/3-HLA* and KIR haplotype study) (solid line). We also included the ROC curves of *IFNL3* (dotted line) and *KIR2DL2/3-HLA* (dashed line) logic model determinations. It can be observed they do not overlap with the best logic regression model. Note: Area under the curve (AUC) of the best model: 0.729 (95% CI, 0.692–0.772).

Logic regression revealed a strong association of the *IFNL3* rs12979860 polymorphism in the prediction of response to combined treatment (PPV = 55.9%) (Table 4). It was improved when we incorporated different studies of KIR combinations in the logic model. The inclusion of the complete analysis of KIR genes and their combinations to the mathematical model yielded the best result (PPV = 75.3%). The ROC curve of different logic regression models was derived. In the best model, the area under the curve (AUC) was 0.729 (95% CI = 0.692–0.772) (Figure 4).

**Table 4:** Prediction of treatment outcome in patients with chronic HCV infection using the proposed logic regression model.

	Sensitivity	Specificity	PPV^3^	NPV^4^	AUC^5^ (95% CI)
**IFNL3** 1	51.7	74.3	55.9	71	0.658 (0.624–0.692)
**KIR2DL2/3-HLA**	16	95.8	70.4	64.5	0.596 (0.575–0.645)
**IFNL3** 1 **+ KIR2DL2/3**	25	93	69	66.3	0.684 (0.642–0.720)
**IFNL3** 1 **+ KIR2DL2/3-HLA**	13.4	97	73.7	64	0.690 (0.651–0.733)
**IFNL3** 1 **+ Haplotypes study**	21.4	94.9	72	65.7	0.699 (0.662.0.735)
**IFNL3** 1 **+ KIR gene study** 2	21.4	95.6	75.3	65.9	0.729 (0.692–0.772)

Note:

1 *IFNL3* polymorphism rs12979860.

2 KIR2DL2/3, KIR2DL2/3-HLA and KIR haplotype study.

3 Positive predictive value.

4 Negative predictive value.

5 Area under the curve.

In conclusion, *KIR2DL3/KIR2DL3-HLA-C1/C1* and the KIR centromeric haplotype motif (A/A genotype) are good predictors of SVR. On the other hand, *KIR2DL2/KIR2DL2-HLA-C1/C2* and the KIR centromeric haplotype motif (B/B genotype) are associated with a high percentage of NSVR in combination with *IFNL3* T/T. Our statistical analysis did not show any association between *KIR2DS2* and treatment outcome.

## Discussion

Previous studies have described several biomarkers of treatment response in addition to *IFNL3* polymorphisms [41], [42]. Other study by our group has described the influence of KIR genes on the progression of chronic HCV infection and treatment outcome [29]. As well as KIR receptors, other genetic markers related to immune response have been reported [42]. One such marker is *PDCD1*, which allowed us to develop a model for predicting the response to conventional therapy. Combining the analysis of this marker with *IFNL3* genotyping considerably improved the predictability of response to standard treatment established using other parameters such as viral load or viral genotype [30].

The current models predict the treatment response of each patient only approximately, so the search for new genetic markers is essential if we are to be able to establish the most appropriate therapy for each patient from the beginning of treatment [30].

As previously reported, factors such as the KIR-HLA genotype were relevant to this response. The *KIR2DL3/KIR2DL3-HLA-C1C1* genotype was more frequent in the SVR group, while the *KIR2DL2/KIR2DL2-HLA-C1C2* genotype was more frequent in NSVR patients. In relation with this, we found that the HLA-C*07 was associated with a good treatment response, especially in combination with *KIR2DL3*. This data is interesting because this HLA-C allele was previously associated with the HCV persistence [43] and, more recently, with high viral load [44].

In order to determine specifically which allelic variant of these genes predominates in the two groups of patients, we carried out an oligotyping analysis of *KIR2DL2* and *KIR2DL3*, as previously described [35], [36]. In relation to *KIR2DL3*, we found that the KIR2DL3*001 allele was most common in SVR while KIR2DL2*001 was more frequent in NSVR. In addition, the relevance of KIR2DL3*001 to good treatment outcome is notably greater when analysed in combination with HLA-C1. These findings suggest these alleles are important in the response to standard treatment, and may be associated with a weak interaction with its ligand, as described for other alleles from *KIR2DL2/3* [45]. Our results confirm the relevance of the *KIR2DL2/3* alleles and the interaction with HLA-C in the treatment outcome, which could also be involved in the progression and chronicity of HCV infection. With regard to *KIR3DL1-HLA-Bw4*, Nozawa et al showed a significant association of this combination with SVR to Peg-IFN/RBV in Japanese population [46]. Nonetheless, we did not find statistical differences in our cohort, in line with previous reports in Caucasian populations. [29], [47]. This data underscore the distinctive organisation of the human KIR locus that drives the generation of KIR gene-content diversity in different populations [48], .

The human KIR locus contains highly conserved genes that are situated in the middle (*KIR3DP1* and *KIR2DL4*) and at the ends (*KIR3DL3* and *KIR3DL2*) of the cluster, creating a framework around two regions of variability in which highly homologous KIR genes are packed very close together in a head-to-tail configuration, separated by short and highly conserved intergenic regions. In different human populations, there is a variable balance between A and B groups of KIR haplotypes, which seems to be maintained by balancing selection for inhibitory and activating functions [50]. This selection is mediated, in part, by the interaction of inhibitory KIR with their HLA class I ligands [51]. A relationship between KIR haplotypes and chronic HCV infection has been demonstrated. Thus, the centromeric KIR haplotype A was increased in patients with resolved HCV infection compared with those who developed a chronic infection [52].

To investigate the role of KIR genes underlying HCV action from a different perspective, in the present study we analysed the relationship between the KIR genotypes and haplotypes with the response to conventional treatment. We found 35 KIR genotypes in our cohort, all of them previously described. The biological significance of the A/B haplotype difference means that it is worth considering whether combinations of A and B haplotypes can influence chronic HCV treatment outcome. The present study has clearly confirmed that such a situation occurs in this series of patients. We found a high frequency of homozygosity for haplotype A in patients with SVR. When we divided this haplotype into the A-centromeric and A-telomeric motifs, we found that the homozygosity for Cen A/A was also more common in SVR patients. This result is controversial because of the high frequency of inhibitory receptors in patients with a good response. This association may have arisen because *KIR2DL3* is present in the Cen A haplotype motif, while *KIR2DL2* is part of Cen B haplotype motif. It has also been shown that *KIR2DL2* and *KIR2DL3* have different inhibitory capabilities arising from dissimilar interactions with their ligands [45]. *KIR2DL1* has the strongest interaction with its ligands, the HLA-C2 alleles, while *KIR2DL2* associates to HLA-C1 more intensely than *KIR2DL3* [53]. Bearing these data in mind, we may surmise that the stronger interaction and therefore the stricter inhibition of NK cells would occur in individuals homozygous for *KIR2DL2* who carry the HLA-C1/C2 genotype. Conversely, NK cells from patients who are homozygous for *KIR2DL3* and for HLA-C1 would have a greater activation ability due to the lack of intense inhibition arising from the interaction between *KIR2DL1* and the HLA-C2 ligand. However, it should be taken into consideration that KIR-activating receptors in the A haplotype are not reduced to *KIR2DS4*, because *KIR2DL4* can sometimes act as an activating receptor [54], [55].

Suppiah et al [56], observed a significant association between HLA groups C1 and C2 with treatment outcome and established a logistic model to predict a failure to clear virus on therapy with PegIFN/RBV. We did not find any association with HLA-C groups and treatment except HLA-C*07. Nonetheless, we observed a clear association between *KIR2DL2/3* and therapy response. Also, we studied all KIR genes to establish the different haplotypes of these receptors, and found a statistical association with SVR. These different results may be due to patient selection and different study population. To assess the significance of these results to treatment outcome, we developed a logic regression model [37], different from Suppiah. This showed that genotyping *IFNL3* alone had a specificity of 74.3% and a positive predictive value (PPV) of 55.9%. However, this result was improved by the determination of KIR genes, increasing the specificity to 95.6% and the PPV to 75.3%. Although the sensitivity decreased from 51.7% to 21.4%, these results are further evidence of the important role that KIR receptors play in the response to combined therapy in HCV-infected patients.

In conclusion, based on our results we propose a novel relationship between KIR/HLA genotypes and KIR haplotypes (A/B) in the chronicity of HCV infection. These could be used as tools in combination with other parameters, such as the determination of *IFNL3* rs12979860, to predict treatment outcome and in order to establish the best cost-effective treatment between the classical regimen and the new arrival therapies. Further research is required to determine the molecular mechanisms involved in this process.

## Supporting Information

Table S1 A) HLA-C alleles distribution in patients with HCV chronic infection. B) KIR2DL2 and KIR2DL3 association with HLA-C*07 allele.
(DOC)
