# Supplementary material for: Diversity of Killer Cell Immunoglobulin-Like Receptor (KIR) Genotypes and KIR2DL2/3 Variants in HCV Treatment Outcome
Source: PLoS One. 2014 Jun 13;9(6):e99426. doi: 10.1371/journal.pone.0099426 (PMC4057177; doi:10.1371/journal.pone.0099426)
Supplement: Table S1 — A) HLA-C alleles distribution in patients with HCV chronic infection. B) KIR2DL2 and KIR2DL3 association with HLA-C*07 allele. (DOC) [file pone.0099426.s001.doc]

Table S1:

A) HLA-C alleles distribution in patients with HCV chronic infection.

| HLA-C | NSVR (2n=996) | SVR (2n=626) | p | OR (95% CI) |
| --- | --- | --- | --- | --- |
| C*01 | 41 (4.1) | 22 (3.5) |  |  |
| C*02 | 53 (5.3) | 33 (5.3) |  |  |
| C*03 | 88 (8.8) | 45 (7.3) |  |  |
| C*04 | 147 (14.8) | 79 (12.6) |  |  |
| C*05 | 94 (9.4) | 44 (7) |  |  |
| C*06 | 81 (8.1) | 40 (6.4) |  |  |
| C*07 | 171 (17.2) | 148 (23.6) | <0.005 | 0.67 (0.52-0.86) |
| C*08 | 62 (6.2) | 38 (6.1) |  |  |
| C*12 | 45 (4.5) | 42 (6.7) |  |  |
| C*14 | 20 (2) | 18 (2.9) |  |  |
| C*15 | 53 (5.3) | 32 (5.1) |  |  |
| C*1507 | 1 (0.1) | 0 |  |  |
| C*1601 | 95 (9.5) | 45 (7.2) |  |  |
| C*1602 | 24 (2.4) | 14 (2.2) |  |  |
| C*17 | 11 (1.2) | 14 (2.2) |  |  |
| C*18 | 10 (1.1) | 12 (1.9) |  |  |

B) KIR2DL2 and KIR2DL3 association with HLA-C*07 allele

| KIR2DL2/3-C*07 | NSVR (n=498) | SVR (n=313) | p | OR (95% CI) |
| --- | --- | --- | --- | --- |
| KIR2DL2-C*07 | 124 (24.9) | 83 (26.5) | NS | - |
| KIR2DL3-C*07 | 136 (27.3) | 141 (45) | <0.001 | 0.46 (0.34-0.62) |

Note: NSVR, non sustained viral responders. SVR, sustained viral responders
